# Generation of HLA Universal Megakaryocytes and Platelets by Genetic Engineering

**DOI:** 10.3389/fimmu.2021.768458

**Published:** 2021-10-27

**Authors:** Constanca Figueiredo, Rainer Blasczyk

**Affiliations:** Institute of Transfusion Medicine and Transplant Engineering, Hannover Medical School, Hannover, Germany

**Keywords:** HLA, megakaryocytes, platelets, gene therapy, gene editing, RNAi

## Abstract

Patelet transfusion refractoriness remains a relevant hurdle in the treatment of severe alloimmunized thrombocytopenic patients. Antibodies specific for the human leukocyte antigens (HLA) class I are considered the major immunological cause for PLT transfusion refractoriness. Due to the insufficient availability of HLA-matched PLTs, the development of new technologies is highly desirable to provide an adequate management of thrombocytopenia in immunized patients. Blood pharming is a promising strategy not only to generate an alternative to donor blood products, but it may offer the possibility to optimize the therapeutic effect of the produced blood cells by genetic modification. Recently, enormous technical advances in the field of *in vitro* production of megakaryocytes (MKs) and PLTs have been achieved by combining progresses made at different levels including identification of suitable cell sources, cell pharming technologies, bioreactors and application of genetic engineering tools. In particular, use of RNA interference, TALEN and CRISPR/Cas9 nucleases or nickases has allowed for the generation of HLA universal PLTs with the potential to survive under refractoriness conditions. Genetically engineered HLA-silenced MKs and PLTs were shown to be functional and to have the capability to survive cell- and antibody-mediated cytotoxicity using *in vitro* and *in vivo* models. This review is focused on the methods to generate *in vitro* genetically engineered MKs and PLTs with the capacity to evade allogeneic immune responses.

## Platelet Transfusion Refractoriness

PLT transfusion refractoriness (PTR) remains a major complication for thrombocytopenic patients due to the high risk for unprompted life-threatening bleeding (1). PTR is characterized by unexpectedly insufficient platelet (PLT) count increments after transfusion. Patients with PLT counts lower than 10x10^9^ show increased predisposition for bleeding. Sixty to 80% of the PLT transfusion refractoriness cases are not associated with immunological aspects, but present non-immune etiologies such as massive bleeding, fever, infection, sepsis, drugs, accelerated PLT consumption, splenic sequestration or graft-versus-host disease (2, 3). Ten to 39% of the cases of PLT transfusion refractoriness are triggered by the development of antibodies specific for antigens expressed on the PLT surface or the development of drug-dependent PLT antibodies (3, 4). Immune causes of PTR include the alloimmunization to the human leukocyte antigen (HLA) and or human PLT antigens (HPA) triggered by previous transfusions, pregnancies, transplantation or PLT autoantibodies. Anti-HLA antibodies are the cause for immune PTR in 90% of the cases (5, 6). This review provides an overview on the strategies to downregulate HLA class I expression on *in vitro* produced Megakaryocytes (MKs) and PLTs as a promising approach to prevent immune PTR due to anti-HLA antibodies.

## HLA Class I Antigens

The HLA system comprises the most polymorphic genes of the entire human genome. HLA class I molecules are constituted by a polymorphic heavy chain non-covalently bound to an invariable beta-microglobulin (β2m) light chain. HLA class I molecules are the protein product of HLA-A, -B and –C genes that are expressed on the cell surface of most nucleated cells (7, 8). HLA class I antigens mainly present endogenous peptides to T-cells and are the basis for a highly desirable and effective anti-viral and anti-tumor cell immune responses. In addition, HLA class I antigens allow the distinction of self- from non-self by providing inhibitory signals to natural killer (NK) cells and therefore supporting the elimination of pathogens and cancer cells. However, after the application of off-the-shelf cell products including cells, tissues or organs, mismatched HLA class I molecules reveal the foreigner identity of the allogeneic cell products by being itself recognized by donor-specific antibodies, alloreactive T-cells or by triggering new humoral or cellular responses (9–12). HLA expression turn PLTs to be highly immunogenic. Multiparous women show HLA sensitization rates up to 74% (13) and despite leukoreduction still 20% of leukemia patients become alloimmunized (14). PLTs express mainly HLA-A and HLA-B on their surface. Accordingly, HLA-C appears to play an irrelevant role in PTR, even though some cases have been described (15). As beta2-microglobulin is a common domain to HLA-A, HLA-B and HLA-C, it has been the selected target to knockdown or knockout the expression of those HLA class I proteins (16).

## Platelets Gene Therapy

Gene therapy is a promising solution for the treatment of diseases by enabling the proper expression of genes in their correct form and adequate level. In particular, monogenetic diseases are a suitable target for treatment based on gene correction or regulation. PLTs are one of the most frequent cells in blood. In addition, PLTs are multifunctional cells that beside the fundamental roles in hemostasis also serve in the storage and delivery of important regenerative factors and regulation of immune responses. PLTs have been and attractive target for gene therapeutic strategies to treat diseases such as hemophilia A, a recessive X-linked bleeding disorder characterized by the factor VIII (FVIII) deficiency. Several studies showed the phenotypic correction of hemophilia A by the ectopic expression of FVIII on PLTs under the control of MK-specific promoters such as the megakaryocytic/PLT-specific glycoprotein IIb (alphaIIb) promoter (17). Also, ectopically expression of Factor IX (FIX) in PLTs showed to correct hemophilia B phenotype (18). Other gene therapeutic strategies as attempt to treat hemophilia A and B have used lentiviral vectors for the delivery of the activated factor Xa precursor gene sequence under integrin αIIb promoter into hematopoietic stem and progenitor cells (19). Hence, such studies have demonstrated the promising values of gene therapy based on PLT engineering.

PLTs are known to regulate important innate and adaptive immune responses such as by inducing recruitment of macrophages, neutrophil autophagy or differentiation and polarization of CD4 T-cells (20–23). Nevertheless, PLTs are also the end target for immune responses that cause their depletion and leading to life-threatening thrombocytopenia. This review is focused on gene therapeutic approaches to generate PLTs with the capacity to evade allogeneic immune responses.

### Genetically Engineered Platelets

Blood pharming is defined by the differentiation of blood cells *in vitro* using protocols that recapitulate hematopoiesis ex vivo. A branch of blood farming technologies is focused on the *in vitro* differentiation of PLTs to serve as an alternative to donor PLTs transfusion.

Several groups have demonstrated the feasibility to differentiate MKs and PLTs *in vitro*. Besides the remarkable value of *in vitro* pharmed PLTs to meet the increasing demand on this product, *in vitro* blood pharming technologies enable the optimization of the differentiated cells towards the decrease of their immunogenicity and thereby potentially increasing their therapeutic efficiency. The generation of genetically engineered MKs and PLTs rely on the selection of an appropriate cell source and method for genetic engineering.

## Cell Sources for Platelets Gene Therapy

The major breakthrough in *in vitro* MK/PLT production was linked with discovery and characterization of thrombopoietin (TPO) and the key role of Mpl receptor in thrombopoiesis in the mid-1990s (24–28). While former attempts to generate primitive MK lines ex vivo have been performed already in the 1980s, the first robust protocol to provide MKs capable of releasing PLTs was developed with introduction of thrombopoietin and published in 1995 (29–31). Several strategies have been explored to improve the quality and quantity of MK/PLT production with the aim to substitute or supplement the demand for donor PLTs in the future therapies.

Several cell sources were evaluated for the capability to differentiate in MKs and PLTs *in vitro*. Initially, CD34+ hematopoietic progenitor and stem cells (HPSCs) were considered the cell type of choice for development of blood pharming protocols and still serve as a golden standard in this field (29, 32, 33). Peripheral blood, umbilical cord blood, bone marrow and fetal liver provided the sources for of CD34+ cell populations with the further capability to differentiate into MKs and PLTS with varied success (29, 31–41). Some attempts have been made to generate MKs from the stromal cells (42–44). While these cell sources provided a fundamental basis for the progress in methods for ex vivo generation of MKs and PLTs, major drawbacks are associated with the use of HPSCs such as their limited proliferation capacity, which leads to continuous necessity of donor material. Thus, a great potential was identified in application of pluripotent cell lines, which are characterized by high proliferative capacity and high plasticity. Embryonic stem cells (ESCs) provide inherent pluripotency and capability to virtually unlimited proliferation *in vitro*, which could have been among the main parameters for the clinically-relevant upscaling of donor-independent MK/PLT production on the basis of this source. Numerous groups developed efficient protocols for differentiation of MKs from ESCs (45–51). However, globally accepted ethical issues with the consequent adaptation of legislative regulations basically limit application of ESCs to the narrow research field and prohibit the progress towards clinics. Shinya Yamanaka and Kazutoshi Takahashi provided a great alternative to ESCs by establishing cellular reprogramming and by introducing a rapid advance in generation of induced pluripotent cell lines (iPSCs) (52). Having similar properties to ESCs, but lacking valid ethical concerns, iPSCs became a major focus in the development of efficient and scalable MK/PLT production protocols. With the first report for successful differentiation of iPSCs into MKs only a decade ago, several progresses have been achieved such as improvement of differentiation efficiency, generation of self-proliferative intermediate MK-progenitor lines, possibility to upscale the production, development of GMP-compatible protocols or biobanking technologies (53–60). Both CD34+ HPSCs and iPSCs were also immediately recognized as promising target for genetic engineering to generate “universal” cell sources for the differentiation of blood products with increased survival after transfusion by escaping allogeneic immune responses.

## Producing MKs and Platelets *In Vitro*

Although the majority of the current methods to ex vivo MK and PLTs production are based on induction with thrombopoietin, several strategies were established to achieve *in vitro* thrombopoiesis (**Table 1**). These methods differ in efficiency, characteristics and functionality of differentiated lines, as well as in time period required for completing the differentiation and maturation. *in vitro* MK and PLT production strategies can be classified into three general approaches: differentiation induced by specific cytokine cocktails, transdifferentiation, and forward programming.

**Table 1:** Strategies to differentiate MKs and PLTs *in vitro*.

Cell source	Medium	Cytokines	Timeline	Reference
CD34+ progenitor cells from peripheral blood	supplemented IMDM	Meg-CSA (natural cytokines contained in protein fraction of aplastic canine serum)	~12-14 days	Mazur et al., 1990 (41)
	serum-free liquid suspension culture medium	IL‐3, IL-3, TPO	~12 days	Guerriero et al., 1995 (31)
	supplemented IMDM	Flt3‐L, IL‐3, TPO	~21 day	Figueiredo et al., 2010 (33)
CD34+ progenitor cells from cord blood	supplemented serum-free IMDM	TPO	~14 days	Tao et al., 1999 (34)
	supplemented serum-free IMDM	SCF, Flt3‐L, IL-6, TPO	~17 days	Proulx et al., 2003 (40)
	SFM	TPO	~12 days	Perdomo et al., 2017 (35)
CD34+ progenitor cells from bone marrow	supplemented serum-free IMDM	TPO	~21 day	Tao et al., 1999 (34)
	serum-free liquid culture system medium	SCF, IL-3, IL-6, G-CSF, TPO	~14 days	Gehling et al., 1997 (36)
	supplemented DMEM with addition of hirudin or heparin	TPO	~5 days	Strassel et al., 2012 (61)
CD34+ progenitor cells from fetal liver	supplemented IMDM	TPO	~12 days	Ma et al., 2000 (37)
	supplemented IMDM	TPO	~5 days	Schulze et al., 2016 (39)
	supplemented DMEM	TPO	~4 days	Vijey et al., 2018 (38)
Embryonic stem cells (ESCs)	supplemented DMEM and αMEM	TPO	~8-16 days	Fujimoto et al., 2003 (51)
	supplemented DMEM and Ham F-12 and IMDM	VEGF, SCF, TPO	~24 days	Takayama et al., 2008 (45)
	supplemented serum-free Stemline II medium	SCF, IL-11, TPO	~14 days	Lu et al., 2011 (47)
Induced pluripotent stem cells (iPSCs)	supplemented mTeSR1, STEMspan-ACF, STEMdiff APEL medium	BMP-4, Flt-3 ligand, IL-3, IL-6, SCF, IL-9, TPO	~19 days	Feng et al., 2014 (60)
	supplemented feeder-free and xeno-free SFM	BMP-4, FGF-2, VEGF, IL-11, SCF, TPO/Nplate	~19 days	Liu et al., 2015 (62)
	StemMACS iPSC brew XF, supplemented APEL 2 medium	BMP-4, VEGF, IL-3, SCF, TPO	~22 days	Eicke et al., 2018 (56)

Cytokine induced differentiation of MK and PLTs as been applied to a wide variety of cell sources. The range, composition, and time points of application of cytokine cocktails are strongly dependent on initial cell type used for differentiation. In the case of initial cells being of hematopoietic origin, such as CD34+, the process basically implies cell culture followed by stimulation with TPO to induce megakaryopoiesis. In the case of pluripotent stem cells, such as ESCs or iPSCs, the process of cytokine-induced MK differentiation includes the following steps: 1) culturing and expansion of initial cell line, 2) mesoderm induction, 3) forwarding the cell fate into hematopoietic progenitors, and, finally, 4) induction of megakaryopoiesis and thrombopoiesis. Cytokine-induced differentiation showed to be a robust method enabling the possibility to upscale the process in bioreactor systems and delivering MKs and PLTs that show functionality *in vivo* (32, 47, 56, 60, 63, 64).

Transdifferentiation is a strategy based on the transformation of one cell type to the other, including processes resembling the switch of stem cells from one tissue type switch to the other (65). *In vitro*, this process can be induced using modern gene engineering tools to promote targeted differentiation of various cell types by direct reprogramming of one cell type to the other circumventing the iPSC stage (66). This approach has been also utilized for transdifferentiation of a range of somatic cell types into MKs. For example, transduction of 3T3 mouse fibroblasts and adult human dermal fibroblasts with nuclear factor erythroid–derived 2 p45 unit and musculoaponeurotic fibrosarcoma oncogene homolog (p45NF-E2/MafG/MafK), followed by culturing in TPO-based MK lineage induction medium, allowed transdifferentiating into MKs with typical morphology and functionality *in vivo* (43, 67). Another group was able to transdifferentiate CD71+ and GPA+ erythroblast subpopulations into functional MKs capable of PLT release by overexpression of the FLI1 and ERG genes (68). Further advances were made by transdifferentiation of embryonic murine fibroblasts and several lines of human skin fibroblasts into MK-like progenitors by overexpression of a set of six transcription factors: LMO2, GATA1, TAL-1, c-Myc, GATA2, and RUNX1 (69). The cells obtained with this method expressed CD41, were polyploid, formed MK colonies, and produced PLTs *in vitro* and engrafted *in vivo*. Moreover, human adipose-derived mesenchymal stromal cells have been transdifferentiated into MKs and PLTs even without the necessity to gene transfer, but with upregulation of endogenous TPO expression by incubation in specific MK lineage induction media (44, 70, 71). Here, the researchers were able to obtain the peak of MK population already at day 8 of cell culture with the peak for PLT release at day 12, also showing functionality *in vivo* in a mouse model (70).

Another key strategy for generating MKs and PLTs *in vitro* is forward programming. The strategy is based on the transient ectopic expression of a range of transcription factors and is mainly applied to pluripotent cell lines (57, 72–74). Successful examples include generation of MKs with minimum cytokines and chemically defined culture by combined expression of GATA1, FLI1, and TAL1 in iPSCs (72). Obtained MKs possessed typical morphology and delivered high purity of population with capability to be maintained in culture for several months (57). Forward programming allows developing GMP-compatible protocols and the generated MKs were capable to withstand cryopreservation procedures, which facilitates biobanking and potential clinical application (57, 72). Moreover, PLT release and purity were improved using bioengineered collagen scaffolds, also showing the importance of the 3D microenvironment (74).

Remarkably, bioreactor engineering for PLT production have focused on optimizing shear stress conditions to increase PLT yield from MKs. Different strategies have been proposed to mimic bone marrow stiffness by using different biomaterials such as collagen-1, collagen-4, fibrinogen, fibronectin, type IV collagen, laminin or alginate. The sinusoidal vessels have been reproduced by silk microtubes or chambers in which specific flows are applied to mimic the shear stress that MKs are exposed to produce PLTs *in vivo*. Also, bottle flasks have been used to produce PTLs using an impeller system to promote the medium flow. This issue has been comprehensively reviewed in Baigger et al. (75–79)

Essential issues such as practicability, quality and safety need to be considered to select the best method of MK/PLT production and ensure translation into clinical application (75). In particular, different methods have been proposed to assess PLT quality including analysis of vitality, morphology, characterization of phenotype (CD34, CD41, CD42a, CD42b, CD49b, CD61) and capacity to aggregate upon stimulation with specific agonists (55, 75, 77, 79–81).

In addition, biodistribution assays have been performed to ensure that *in vitro* produced and genetically modified MK/PLTs transfused into mouse models do not accumulated in specific tissues and organs. Remarkably, irradiation of *in vitro* generated MKs did not affect PLT production and may represent an effective strategy to support safety of this iPSC-derived cell products (55, 56, 63).

## Strategies for Genetic Engineering

Different strategies have been developed to genetically engineer cell sources for MK and PLT production *in vitro* (**Table 2**).

**Table 2:** Impact of regulation of HLA expression on MKs and PLTs in functionality and survival to allogeneic immune responses.

Cell source	Targeted Gene	Genetic engineering technology	Function Test of genetically engineered PLTs	Survival to antibody mediated cytotoxicity	No target to NK cell cytotoxicity	Reference
MK-iPSC	B2m	CRISPR/Cas9	*In vitro* aggregation	*In vivo*	*In vitro* and *In vivo*	Suzuki et al., 2020 (82)
IPSCs	B2m	CRISPR/Cas9 nickases	*In vitro* activation and aggregation	Not evaluated	Not evaluated	Norbnop et al., 2019 (83)
IPSCs	B2m	RNAi/shRNA	*In vitro* activation	*In vitro* and *in vivo* survival	Not evaluated	Börger et al., 2016 (55)
IPSC	B2m	TALEN	*In vitro* activation	Not evaluated	Not evaluated	Feng et al, 2014 (60)
HPSC	B2m	RNAi/shRNA	*In vitro* activation and aggregation	*In vitro* and *in vivo*	Not evaluated	Gras et al., 2013 (63)
HPSC	B2m	RNAi/shRNA	*In vitro* activation and aggregation	*In vitro*	Not evaluated	Figueiredo et al., 2010 (33)

### Gene Regulation

After the discovery of RNAi interference (RNAi) in 1998 by Craig and Mello (84) showing its potential as promising tool for the development of cell and gene-therapeutic approaches, RNA interference is a gene regulatory mechanism that prevents protein translation by activating sequence-specific RNA degradation. Hence, RNAi is a highly specific and selective process enabling the control of protein expression. RNAi is initiated by double strand RNA (dsRNA) that after being processed and integrated into a multimeric protein complex lead to the sequence-specific degradation of a target mRNA. First, the RNase III Dicer cleaves the long dsRNA into 21-23-nucleotide small interfering RNA (siRNA). Then, the siRNA is incorporated in the RNA-inducing silencing complex (RISC) which is associated with the Argonaute 2 (Ago2) cleaving enzyme. The helicase activity of RISC unwind the siRNA allowing one of the siRNA strands to serve as guiding RNA sequence to RISC/Ago2 for the recognition of the target mRNA. After hybridization with the target mRNA, the RISC/Ago2 complex promotes the target mRNA cleavage which causes post-transcriptional gene silencing (85). RNAi has been widespread used in proof-of-concept studies and clinical trials (86). In previous studies, we have shown the feasibility to silence HLA class I expression on the surface of MKs and PLTs by lentiviral vector-mediated transduction of CD34+ HPSCs or iPSCs for expression of shRNAs targeting β2-microglobulin transcripts (33, 55, 63). Silencing HLA class I on the progenitor/stem cells did not impair their capacity to be differentiated into MKs and PLTs. In iPSCs, the expression of HLA class I antigens was silenced by up to 82%. The HLA class I silencing effect was maintained during several passages of iPSC culture. iPSC culture and differentiation was performed under feeder-and xeno-free conditions. HLA class I-silenced MKs showed typical morphology and increase of ploidy. HLA class I silenced PLTs were capable to upregulate the expression of activation markers such as CD62P and to aggregate upon stimulation with ADP and thrombin, indicating that HLA silencing does not impair the functionality of PLTs. Remarkably, in contrast to MKs and PLTs derived from fully HLA class I expression cell sources, HLA class I-silenced MK and PLTs showed significantly lower lysis rates in *in vitro* complement-dependent cytotoxic assays as well as antibody mediated cellular-dependent cytotoxic assays suggesting that HLA class I silenced MK and PLTs are capable to survive under refractoriness conditions. This was confirmed using *in vivo* PLT transfusion refractoriness mouse model showing that HLA class I-silenced MKs and PLTs prevail in the mouse circulation for longer periods than HLA-expressing MK/PLTs (55, 63). Hence, the use of RNA interference to downregulate HLA class I expression on MKs and PLTs demonstrated to be an efficient strategy to decrease their immunogenicity and improve cell survival after application in PLT transfusion refractory recipients. The use of RNAi to silence HLA class I expression is associated with intrinsic advantages and disadvantages. The efficiency of the delivery of the RNAi cassettes encoding for shRNA using lentiviral vectors into the MK/PLT cell source is depending on the type of the cell. Although the efficiency of the genetic modification of CD34+ cells is moderate to low, it is very high in high proliferative cell sources such as iPSCs. Furthermore, it is necessary to monitor the silencing effect during the culture of the cell source and during differentiation to ensure a sufficient HLA-silencing effect (33, 55). The residual HLA class I expression may be beneficial to prevent undesired NK cell cytotoxicity towards HLA class I-silenced MKs after transfusion.

### Gene Editing

Site-specific gene editing technologies have also been used in PLT gene therapy and are mainly based on the use of Transcription-Activator Like Effector Nucleases (TALEN) and Clustered Regularly Interspaced Short Palindromic Repeats (CRISPR)-Associated 9 (CRISPR-Cas9) including its variants such as nickase Cas9 (60, 82, 83). Targeted genome editing relies on the generation of nuclease-induced double-stranded breaks (DSBs) which support highly efficient recombination mechanisms of DNA. Nuclease-induced DNA DSBs can be repaired by homology-directed repair (HDR) and nonhomologous end-joining (NHEJ) resulting in targeted integration or gene disruptions, respectively.

TALENs result from the fusion of transcription activator-like (TAL) proteins with a FokI nuclease. TAL proteins are composed of 33-35 amino acid repeating motifs with two variable positions that recognize single nucleotides. Hence, TALENs induce DSBs into specific DNA sites, which are then repaired (87). In 2014, Feng and colleagues have disrupted the β2-microglobulin gene to generate HLA class I knockout iPSCs under feeder- and xeno-free conditions. Furthermore, they show the feasibility to generate HLA class I knockout MK and PLTs from those source. The differentiated PLTs showed to respond *in vitro* to thrombin in PAC-1 binding assays (60). Suzuki and colleagues have knockout β2m using CRISPR/Cas9 nuclease technology. The group has re-reprogramed the previously immortalized MK cell lines (imMKCLs) into iPSCs (MK-iPSCs) prior the generation of β2m knockout. The group confirmed that the generated MK-iPSCs without HLA class I expression still carry the DOX-inducible c-MYC, BCL-XL and BMI1 transgenes of the original iMKCL and also could be re-induced, expanded and used for PLT production (iPLATs). Furthermore, the authors showed *in vitro* that the HLA KO iPLATs were not a target for NK cell cytotoxicity. They have confirmed those results *in vivo* using a NK cell reconstituted humanized mice treated with anti-HLA-A2 antibodies. The group propose that NK cell tolerance against HLA KO iPLATs might be due to the absence of NK cell activating ligands expression (82). In 2013, Ran et al. proposed the use of a D10A mutant nickase version of Cas9 with a pair of guide RNAs complementary to opposite strands of the target to generate DSBs. The group shows that individual nicks may increase genome editing specificity (88). Therefore, Norbnop and colleagues proposed the use of CRISPR/Cas9 nickase strategies to knockout β2m in iPSCs to be used as cell source for PLTs. After genetic modification, the iPSCs presented a normal karyotype. They differentiate β2m KO CD34+ HSCs from iPSCs using culture with 10T1/2 feeder cells. Isolated HLA KO iPSC-derived HSCs were used to differentiate MKs which showed typical proPLT and PLT formation. β2m KO PLTs showed to respond *in vitro* to thrombin with the upregulation of CD62P expression (83). These studies showed the feasibility to generate gene edited HLA-universal PLTs with the capacity to survive alloimmune responses. The efficiency of editing HLA may vary depending on the cell type and on the strategy to bring the gRNA and enzymes into the target cell. In case of bi-allelic gene editing, the cells will completely lack HLA class I expression, which may completely prevent the binding of anti-HLA class I antibodies.

Genetic engineering of MKs and PLTs using the above mentioned technologies is associated with the risk for off-target effects caused by unspecific cutting which may create undesired mutations. Several approaches have been designed to improve gRNA design and increase the specificity of the Cas. Current studies have demonstrated the absence or only rare off-target sites, long-size deletions or chromossomal rearrangements were observed. In addition, the number of such off-target effects has decreased with time. Nevertheless, precise methods including high-throughput sequencing and multi-omics analyses are highly desirable to detect sporadic off-target effects. Also, anti-Cas antibodies and anti-Cas T-cells have been detected (89, 90). Blood pharming supports the safe application of genetic engineered products, as the target for genetic engineering is cell source (e.g. iPSC line) and not the applied product (e.g. PLTs). Hence, there is the time and possibility for the completely characterization and evaluation of potential off-target effects including the formation of neo-antigens that could trigger unwanted immune responses towards the transfused PLTs. Only the genetically engineered iPSC that show no off-target effect will be selected for *in vitro* production of MKs and PLTs. This has the potential to abrogate major safety concerns.

## Conclusion

The feasibility to improve the therapeutic effect of *in vitro* generated MKs and PLTs has laid the foundation for the “next generation” of blood pharming. Genetic engineering may not only be applied to facilitate the *in vitro* differentiation of PLTs from different cells sources, but also to regulate the expression of antigens on their surface such as HLA class I and thereby supporting their survival after transfusion. The translation of this technology into clinical application is tightly associated with the development of effective bioreactors and production strategies that allow the fabrication of enough numbers of MKs and PLTs as well as reducing the production costs. HLA universal PLTs may become an important biotherapeutic product for the treatment of severe immunized thrombocytopenic patients.

## Author Contributions

CF and RB: conception, design, and writing of the manuscript. All authors contributed to the article and approved the submitted version.

## Funding

We acknowledge the Foundation of the German Red Cross - Service of blood donation NSTOB (Project: StiBSD/V2871) for funding part of the authors’ work mentioned in this review.

## Conflict of Interest

The authors declare that the research was conducted in the absence of any commercial or financial relationships that could be construed as a potential conflict of interest.

## Publisher’s Note

All claims expressed in this article are solely those of the authors and do not necessarily represent those of their affiliated organizations, or those of the publisher, the editors and the reviewers. Any product that may be evaluated in this article, or claim that may be made by its manufacturer, is not guaranteed or endorsed by the publisher.
